# Gut microbiota and metabolomic profile changes play critical roles in tacrolimus-induced diabetes in rats

**DOI:** 10.3389/fcimb.2024.1436477

**Published:** 2024-09-17

**Authors:** Zhenwei Jiang, Minyan Qian, Zeng Zhen, Xuping Yang, Caomei Xu, Li’an Zuo, Jingting Jiang, Wenting Zhang, Nan Hu

**Affiliations:** ^1^ Department of Pharmacy, The Third Affiliated Hospital of Soochow University/The First People’s Hospital of Changzhou, Changzhou, China; ^2^ Changzhou Key Laboratory of Human Use Experience Research & Transformation of Menghe Medical School, Changzhou Hospital Affiliated to Nanjing University of Chinese Medicine, Changzhou, China; ^3^ Department of Tumor Biological Treatment, The Third Affiliated Hospital of Soochow University, Changzhou, China; ^4^ Pediatric Central Laboratory, Affiliated Changzhou Children’s Hospital of Nantong University, Changzhou, China

**Keywords:** tacrolimus, diabetes, gut microbiota, metabolomics, amino acids, bile acids, short chain fatty acids

## Abstract

**Aims:**

Hyperglycemia is one of the adverse effects of tacrolimus (TAC), but the underlying mechanism is not fully identified. We used multi-omics analysis to evaluate the changes in the gut microbiota and metabolic profile of rats with TAC-induced diabetes.

**Methods:**

To establish a diabetic animal model, Sprague Dawley rats were divided randomly into two groups. Those in the TAC group received intraperitoneal injections of TAC (3 mg/kg) for 8 weeks, and those in the CON group served as the control. 16S rRNA sequencing was used to analyze fecal microbiota. The metabolites of the two groups were detected and analyzed by nontargeted and targeted metabolomics, including amino acids (AAs), bile acids (BAs), and short-chain fatty acids (SCFAs).

**Results:**

The rats treated with TAC exhibited hyperglycemia as well as changes in the gut microbiota and metabolites. Specifically, their gut microbiota had significantly higher abundances of *Escherichia-Shigella*, *Enterococcus*, and *Allobaculum*, and significantly lower abundances of *Ruminococcus*, *Akkermansia*, and *Roseburia*. In addition, they had significantly reduced serum levels of AAs including asparagine, aspartic acid, glutamic acid, and methionine. With respect to BAs, they had significantly higher serum levels of taurocholic acid (TCA), and glycochenodeoxycholic acid (GCDCA), but significantly lower levels of taurodeoxycholic acid (TDCA) and tauroursodeoxycholic acid (TUDCA). There were no differences in the levels of SCFAs between the two groups. Correlations existed among glucose metabolism indexes (fasting blood glucose and fasting insulin), gut microbiota (*Ruminococcus* and *Akkermansia*), and metabolites (glutamic acid, hydroxyproline, GCDCA, TDCA, and TUDCA).

**Conclusions:**

Both AAs and BAs may play crucial roles as signaling molecules in the regulation of TAC-induced diabetes.

## 1 Introduction

Tacrolimus (TAC) is a macrolide antibiotic produced by the soil fungus *Streptomyces tsukubaensis*. It is the most widely used calcineurin inhibitor for organ transplant patients and is frequently used to prevent organ rejection after transplantation. It is characterized by a narrow therapeutic index and high inter- and intra-patient pharmacokinetic variability, and the use of excessive TAC is associated with nephrotoxicity, diabetes, opportunistic infections, increased cardiovascular risk, and malignancies. However, insufficient TAC may result in the rejection and eventually the loss of the graft (Brunet et al., 2019; Degraeve et al., 2020). Research has shown that the prolonged use of TAC can lead to hyperglycemia, which is a significant risk factor for post-transplant diabetes mellitus (PTDM) (Jouve et al., 2018). As early as 1987, Lewis rats were found by Nalesnik et al. to exhibit signs of elevated blood glucose following long-term TAC administration (Nalesnik et al., 1987). While thoroughly understanding the pathogenic mechanism underlying TAC-induced diabetes is crucial for managing PTDM, existing research is largely limited to the toxic effect of TAC on islet β cells. Indeed, TAC can induce diabetes by affecting the signaling pathways related to the maintenance and function of β cells (Rodriguez-Rodriguez et al., 2021; Tong et al., 2021), such as calcineurin (CaN)/nuclear factor of activated T cells (NFAT) (Goodyer et al., 2012), phosphatidylinositol 3-kinase (PI3K)/protein kinase B (AKT)/mammalian target of rapamycin (mTOR) (Rodriguez-Rodriguez et al., 2019), transforming growth factor-β (TGF-β)/mothers against decapentaplegic homolog (Smad) (Chen et al., 1997), and tyrosine-protein kinase (Syk)/B-cell linker protein (BLNK)/nuclear factor-κB (NF-κB) (Chen et al., 2024).

Gut microbiota is a very complex microbial ecosystem that exists in gastrointestinal tract and is considered a special metabolic “organ” of the body (Huda et al., 2021). Thanks to the advance of 16S rRNA and metagenome sequencing technology, the influence of the gut microbiota on type 2 diabetes mellitus (T2DM), including its participation in glucose regulation and insulin sensitivity, has been recognized (Fu et al., 2018; Gurung et al., 2020). Some studies also reported that TAC can alter the composition and bacterial taxa of the gut microbiota (Zhang et al., 2018). Meanwhile, TAC-induced glucose metabolism disorders can be aggravated by altering the gut microbial composition and function (Han et al., 2019). Presumably, the gut microbiota participates in the process of TAC-induced diabetes.

The regulation of glucose homeostasis by the gut microbiota depends on multiple metabolites and their interactions with host cell receptors, including short-chain fatty acids (SCFAs), amino acids (AAs), bile acids (BAs), and so on (Zhai et al., 2021; Zheng et al., 2021). Studies have demonstrated that SCFAs can promote the secretion of glucagon-like peptide-1 (GLP-1) by activating free fatty acid receptor 2 (FFAR2), which indirectly modulates blood glucose levels by enhancing insulin secretion and reducing glucagon secretion (He et al., 2020). In terms of branched-chain AAs (BCAAs), both *in vivo* and *in vitro* studies have indicated that decreased BCAA levels can improve insulin sensitivity by inhibiting the mTOR/S6K1 signaling pathway, thereby relieving the inhibition of the insulin signaling pathway (Zhu et al., 2016). In addition, BAs can regulate glucose homeostasis by directly interacting with the farnesoid X receptor (FXR) and the G protein-coupled bile acid receptor 1 (TGR5) in the liver, intestine, and pancreas. They also indirectly stimulate FXR-dependent intestinal fibroblast growth factor 15 (FGF15/19) production (Sah et al., 2022). Metabolomics reveals the link between genotype and phenotype in biological systems. By quantifying the changes in the absolute and/or relative amounts of thousands of small molecule metabolites in blood and tissues, it provides valuable insights into disease diagnosis, pathogenesis, and drug intervention (Ribbenstedt et al., 2018). Several metabolomic methods have been developed to evaluate biomarkers of diabetes (Schrimpe-Rutledge et al., 2016; Hu et al., 2021). However, there are limited studies on the changes in the gut microbiota and the related metabolites in TAC-induced diabetic models (Canfora et al., 2019; Li et al., 2024).

In this study, to screen markers related to TAC-induced diabetes, we used 16S rRNA gene sequencing to analyze the changes in the gut microbiota of TAC-induced diabetic rats and nontargeted/targeted metabolomics to detect the serum metabolic profiles. In addition, we further analyzed the correlation between the gut microbiota, metabolites, and the glucose metabolism indexes and discussed the mechanism of TAC-induced diabetes from a new angle.

## 2 Materials and methods

### 2.1 Materials

TAC was purchased from Popeye Biotechnology (Shenzhen) Co., Ltd. (Shenzhen, China). Methanol, acetonitrile, formic acid, and ammonia were supplied by Merck (Dannstadt, Germany). Methoxyamine HCl, fatty acid methyl esters, and pyridine were provided by Sigma-Aldrich (St. Louis, MO, USA). *N*-Methyl-*N*-(trimethylsilyl)trifluoroacetamide (MSTFA), hexane, methylene chloride, and chloroform were purchased from Thermo-Fisher Scientific (Fair Lawn, NJ, USA). Bile acid (BA) standards, including cholic acid (CA), glycocholic acid (GCA), deoxycholic acid (DCA), chenodeoxycholic acid (CDCA), ursodeoxycholic acid (UDCA), glycochenodeoxycholic acid (GCDCA), taurocholic acid (TCA), tauroursodeoxycholic acid (TUDCA), glycoursodeoxycholic acid (GUDCA), taurochenodeoxycholic acid (TCDCA), taurodeoxycholic acid (TDCA), lithocholic acid (LCA), and taurolithocholic acid (TLCA), and isotope internal standards were supplied by Sigma-Aldrich (St. Louis, MO, USA). Deionized water was generated in the lab using a Milli-Q system (EMD Millipore, Danvers, MA, USA).

### 2.2 Animal experiment and sample collection

Eight-week-old male Sprague Dawley (SD) rats (180-220 g) were supplied by Cavens Experimental Animal Co., Ltd. (Changzhou, China). The rats were kept in a standard specific pathogen free (SPF) environment with a 12 h/12 h light/dark cycle and acclimatized for 7 days before the experiment. The rats were randomly divided into the control group (CON) and the tacrolimus group (TAC), with six rats in each group, and received intraperitoneal injections of either 3 mg/kg/d TAC (TAC group) or an equal volume of normal saline (CON group) for 8 weeks. After 8 weeks, the feces, the serum, and the pancreatic tissue were collected. The experiment protocol complied with the institutional guidelines on the care and use of laboratory animals was certified by the Ethics Committee of the Third Affiliated Hospital of Soochow University (Approval No. 2021151).

### 2.3 Fasting blood glucose and oral glucose tolerance test

Blood samples were collected from the caudal vein after the rats were fasted for 12 h with free access to water. The FBG was measured using an Accu-Chek Active blood glucose meter. For the OGTT, the rats were given 40% glucose (2 g/kg body weight) via gavage, and the blood samples collected at 0, 30, 90, 120, and 180 min after gavage were analyzed.

### 2.4 Biochemical indicators and serum insulin

An AU5800 automatic biochemistry analyzer (Beckman Coulter, Brea, CA, USA) was used to analyze the serum biochemical parameters. The serum insulin levels were determined using ELISA kits (YX-091419R, Yuanxin Biotechnology, Shanghai, China). The homeostasis model assessment was used to calculate the following indexes from the FBG (mmol/L) and the fasting insulin (FINS, mU/L):


$$\text{HOMA}-\text{IR}=\text{FBG}\times \frac{\text{FINS}}{22.5}$$

$$\text{HOMA}-\text{β}=20\times \frac{\text{FINS}}{\text{FBG}-3.5}$$

$$\text{HOMA}-\text{ISI}=\frac{1}{\log\text{FBG}+\log\text{FINS}}$$

where HOMA-IR is the insulin resistance index, HOMA-β is the insulin secretion index, and HOMA-ISI is the insulin sensitivity.

### 2.5 Histological assessment

The pancreatic damage was assessed using hematoxylin-eosin (H&E) staining according to a published protocol (Hu et al., 2021). The stained slides were observed and photographed under an Olympus CX31 microscope (Olympus, Hamburg, Germany).

### 2.6 Gut microbiota analysis

The microbial DNA in the collected feces was extracted using the E.Z.N.A.^®^ Stool DNA Kit (Omega Biotek, Norcross, GA, USA) according to the manufacturer’s instructions. The V3-V4 region of the 16S rRNA gene of the bacteria was amplified on a GeneAmp 9700 thermocycler (Applied Biosystems, Waltham, MA, USA) using the primers 338F (5’-ACTCCTACGGGAGGCAGCAG-3’) and 806R (5’-GGACTACHVGGGTWTCTAAT-3’). The structure of the gut microbiota was constructed after amplification and sequencing on the Illumina MiSeq platform, and the abundance of the OTUs was determined using USEARCH 7.17.1 (http://drive5.com/uparse/).

### 2.7 Non-targeted and targeted metabolomics

#### 2.7.1 Non-targeted metabolomics analysis

Serum samples were thawed in an ice bath and centrifuged at 4°C and 3,000 *g* for 5 min. A 50 μL aliquot of the serum was added to precooled methanol/chloroform (3/1 v/v, 175 μL) and L-2-chlorophenylalanine (10 μL, internal standard). The mixture was centrifuged at 4°C and 14,000 *g* for 20 min. A 200 μL aliquot of the supernatant was freeze-dried, and the supernatant of all remaining samples was mixed in equal volume to give a quality control (QC) sample. To the residue was added an aqueous solution of methoxylamine hydrochloride pyridine (20 mg/mL, 50 μL), and the mixture was maintained at 30°C for 2 h before it was derivatized with FAMEs (50 μL) containing MSTFA (1% TMCS) at 37.5°C for 60 min and analyzed by GC-MS (Wu et al., 2021).

The obtained raw GC-MS data files were processed using Chroma TOF (v4.71, Leco Corp., St. Joseph, MO, USA) for baseline denoising, smoothing, peak extraction, deconvolution, and peak alignment. Compounds were identified by comparing the MS similarity and the FAMEs retention index distance using the JiaLib database. The data were imported into iMAP (Metabo-Profile, Shanghai, China) for multivariate statistical analysis, including principal component analysis (PCA) and orthogonal partial least squares discriminant analysis (OPLS-DA). The metabolites were defined as differential metabolites if their variable importance in the projection (VIP) value in the OPLS-DA model was greater than 1 and they showed statistically significant difference between the TAC and CON groups. The reliability and prediction ability of the model were assessed with *R^2^* (the goodness of fit parameter) and *Q^2^* (the goodness of prediction parameter). The enrichment analysis and topological analysis of the differential metabolites were performed using the MetaboAnalyst software (https://www.metaboanalyst.ca/).

#### 2.7.2 Targeted metabolomics analysis

Serum BAs were determined according to the validated high-performance liquid chromatography tandem mass spectrometry (HPLC-MS/MS) method reported in the literature (Hu et al., 2021). Fecal BAs were determined as follows. The feces sample was sonicated in methanol (9 mL per gram fecal sample) for 30 min, and the mixture was then centrifuged at 16,400 rpm for 20 min. To an aliquot of the supernatant (50 μL) was added the internal standard (20 μL) and acetonitrile (200 μL). The internal standard contained GCA-d5, CDCA-d4, GCDCA-d7, DCA-d5, and LCA-d4, all at 100 ng/mL. The mixture was vortexed for 30 s and then centrifuged at 16,400 rpm for 10 min. The supernatant was diluted with an equal volume of ultrapure water, transferred to a glass vial, and analyzed by HPLC-MS/MS.

To measure the serum AAs, the serum (50 μL) was vortexed in 50% acetonitrile (200 μL), and the mixture was centrifuged at 4°C at 16,400 rpm for 10 min. The supernatant was centrifuged again at 4°C at 16,400 rpm for 5 min, and the serum AAs were then determined by HPLC-MS/MS.

For the analysis of serum SCFAs, the serum (50 μL) was added into acetonitrile (100 μL) using a pipette, and the mixture were ultrasonicated at 5°C and 40 kHz for 30 min before centrifugation at 4°C and 13,000 rpm for 15 min. To the supernatant was added the aqueous solutions of 3-NPH·HCl (200 mM, 20 μL) and EDC·HCl (120 mM and containing 6% pyridine, 20 μL). The mixture was then reacted at 40°C for 30 min, diluted with 50% acetonitrile to 750 μL, and analyzed by UPLC-MS/MS.

The detailed chromatographic and mass spectrometric conditions for non-targeted and targeted metabolomic analysis are given in **Supplementary Tables S1–S6**.

### 2.8 Statistical analysis

Data were expressed as mean ± standard deviation, and SPSS 25.0 was used for statistical analysis. Statistically significance (*P*< 0.05) in the difference between two groups was verified by the Student’s t test for normally distributed continuous variables and by the Mann-Whitney U test for non-normally distributed variables. The Spearman correlation analysis was used to analyze the correlation among gut microbiota, BAs, AAs, and physiological data.

## 3 Results

### 3.1 Establishment of TAC-induced diabetic rat model

**Figure 1:**
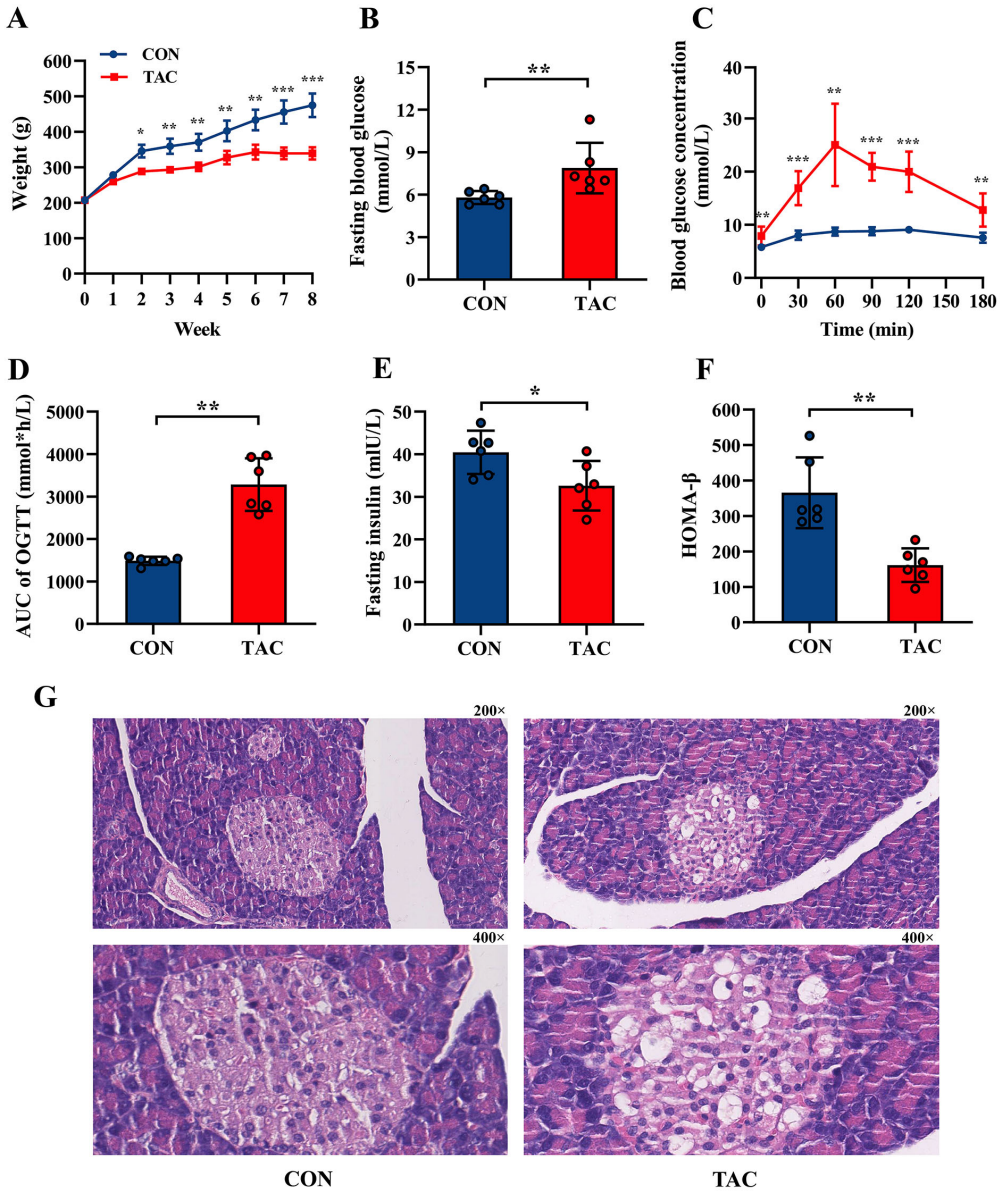
Effects of TAC on SD rats. **(A)** Body weight; **(B)** Fasting blood glucose; **(C)** Oral glucose tolerance test (OGTT); **(D)** Area under curve (AUC) of OGTT; **(E)** Fasting insulin; **(F)** HOMA-β index; **(G)** Pancreatic histological structure with 200× and 400× magnification, respectively. *P< 0.05, **P< 0.01, ***P< 0.001.

After 8 weeks, the rats in the TAC group had much lower body weight than the rats in the CON group, and their FBG and area under the curve (AUC) of OGTT were significantly higher (**Figures 1A–D**). The TAC group had significantly lower FIN and HOMA-β than the CON group (**Figures 1E, F**), but there was no difference in HOMA-IR and HOMA-ISI between the two groups (**Supplementary Figure S1**). In the histological assessments, compared to the rats in the CON group, the rats in the TAC group had fewer the islet cells, many vacuoles appeared in the cytoplasm of their islet cells, and the nuclear staining was uneven (**Figure 1G**). Apparently, the TAC treatment destroyed islet cells and reduced insulin secretion.

In addition, compared to the CON group, the TAC group had significantly higher TC, HDL-C, and urea levels, as well as significantly lower AST, LDH, Na^+^, Cl^−^ and Mg^2+^ levels. No difference was observed in the TG, LDL-C, Cr, ALT, ALP, GGT, TP, ALB, GLB, and UA levels between the two groups (**Table 1**).

**Table 1:** Biochemical parameters.

Parameters	CON	TAC
TC (mmol/L)	2.10 ± 0.32	2.61 ± 0.27*
TG (mmol/L)	1.78 ± 0.68	1.46 ± 0.62
HDL-C (mmol/L)	1.62 ± 0.31	2.09 ± 0.31*
LDL-C (mmol/L)	0.39 ± 0.07	0.46 ± 0.06
Urea (mmol/L)	4.96 ± 0.28	8.85 ± 1.14***
Cr (μmol/L)	30.83 ± 2.19	33.67 ± 2.56
ALT (U/L)	44.25 ± 7.45	39.12 ± 5.71
AST (U/L)	121.68 ± 17.21	65.80 ± 25.21**
ALP (U/L)	234.50 ± 32.56	233.83 ± 66.33
GGT (U/L)	0.13 ± 0.18	0.65 ± 0.68
LDH (U/L)	1014.83 ± 321.25	486.00 ± 236.50*
TP (g/L)	74.03 ± 2.59	70.15 ± 5.64
ALB (g/L)	36.22 ± 1.21	34.62 ± 2.24
GLB (g/L)	37.82 ± 1.83	35.53 ± 1.26
UA (μmol/L)	127.22 ± 25.29	122.60 ± 33.5
K^+^ (mmol/L)	5.26 ± 0.25	4.67 ± 1.26
Na^+^ (mmol/L)	150.58 ± 0.86	147.67 ± 2.62*
Cl^-^ (mmol/L)	102.27 ± 0.43	96.63 ± 1.23***
Ca^2+^ (mmol/L)	2.63 ± 0.04	2.71 ± 0.71
Mg^2+^ (mmol/L)	1.06 ± 0.06	0.53 ± 0.08*

Values are expressed as mean ± standard deviation; TC, total cholesterol; TG, triglycerides; HDL, high-density lipoprotein; LDL, low-density lipoprotein; Cr, creatinine; ALT, alanine aminotransferase; AST, glutamic oxaloacetic transaminase; ALP, alkaline phosphatase; GGT, gamma-glutamyltransferase; LDH, lactic dehydrogenase; TP, total protein; ALB, albumin; GLB, globulin; UA, uric acid; *P< 0.05, **P< 0.01, ***P< 0.001.

### 3.2 Effects of TAC on composition of the gut microbiota

**Figure 2:**
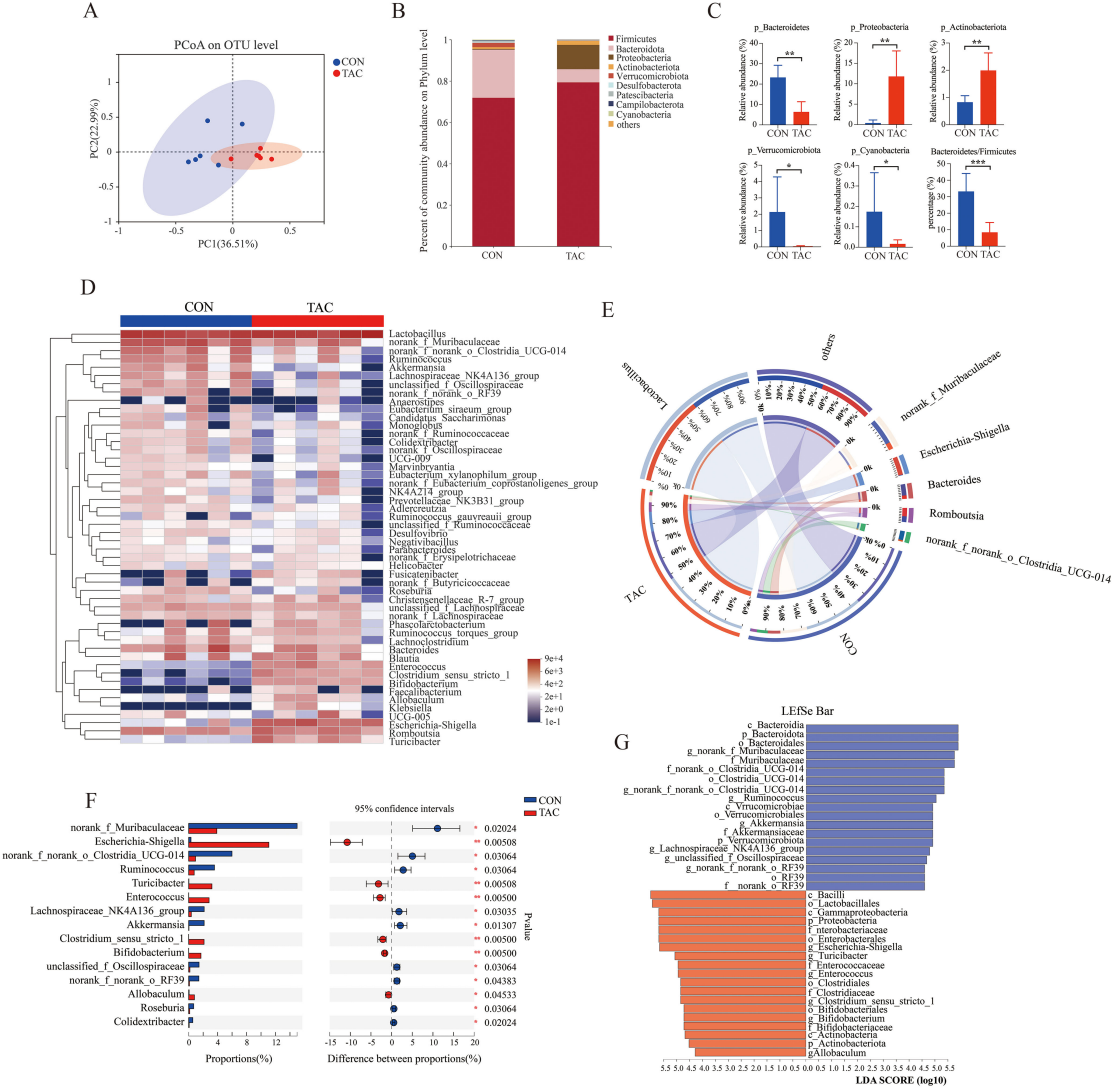
Effects of TAC on the structure of the gut microbiota. **(A)** PCoA based on OTU; **(B)** Relative abundance of gut microbiota at the phylum level; **(C)** The relative abundance of key phyla in the gut microbiota; **(D)** Heatmap of the relative abundance of the top 50 dominant genera; **(E)** Community Circos diagram; **(F)** The relative abundance of different microbiota at the genus level; **(G)** LEfSe data (LDA > 4.0; *P< 0.05, **P< 0.01, ***P< 0.001.

In the principal co-ordinates analysis (PCoA), the fecal samples of the two groups were divided into different clusters, indicating that TAC changed the composition of the gut microbiota in rats (**Figure 2A**). The Chao1, ACE, and Shannon indexes were lower in the TAC group than in the CON group, but the differences were not statistically significant (**Supplementary Table S7**). At the phylum level, the TAC group had significantly higher relative abundances of *Proteobacteria* and *Actinobacteriota* and lower relative abundances of *Bacteroidetes*, *Verrucomicrobiota*, and *Cyanobacteria*. The ratio of *Bacteroidetes* to *Firmicutes* was significantly lower in the TAC group (**Figures 2B, C**).

The top 50 differentiated taxa with the highest genus level could be extracted from the heatmap (**Figure 2D**). The relatively dominant taxa were *Lactobacillus*, *norank_f_Muribaculaceae*, and *Bacteroides* in the CON group but *Lactobacillus*, *Escherichia-Shigella*, and *Romboutsia* in the TAC group (**Figure 2E**). With reference to the CON group, the relative abundances of *Escherichia-Shigella*, *Turicibacter*, *Enterococcus*, *Bifidobacterium*, and *Allobaculum* were much higher, and the relative abundances of *Ruminococcus*, *Akkermansia*, *Colidextribacter*, and *Roseburia* were much lower in the TAC group (**Figure 2F**). The linear discriminant analysis (LDA) effect size (LEfSe) was used to further identify different taxa that may be microbiological markers. With the LDA score cutoff at 4.0, the following key enrichments were noted (**Figure 2G**): in the CON group, *p_Bacteroidota*, *o_Bacteroidales*, *c_Bacteroidia*, *o_Clostridia_UCG-014*, *c_Verrucomicrobiae*, and *f_Akkermansiaceae*; in the TAC group, *c_Bacilli*, *o_Lactobacillales*, *c_Gammaproteobacteria*, and *p_Proteobacteria*.

We further ran the correlation analysis between the differential gut genera and the physiological data. The FBG and the AUC of OGTT were correlated positively with the relative abundances of *Escherichia-Shigella*, *Enterococcus*, and *Bifidobacterium* but negatively with the relative abundances of *Ruminococcus*, *Roseburia*, and *Akkermansia*. The FIN and HOMA-β levels were correlated positively with *Akkermansia* but negatively with *Enterococcus*. The changes in TG, TC, and HDL-C could also be associated with the gut microbiota (**Supplementary Figure S2**). The results suggested that the changes in the gut microbiota likely played an important role in TAC-induced diabetes.

**Figure 3:**
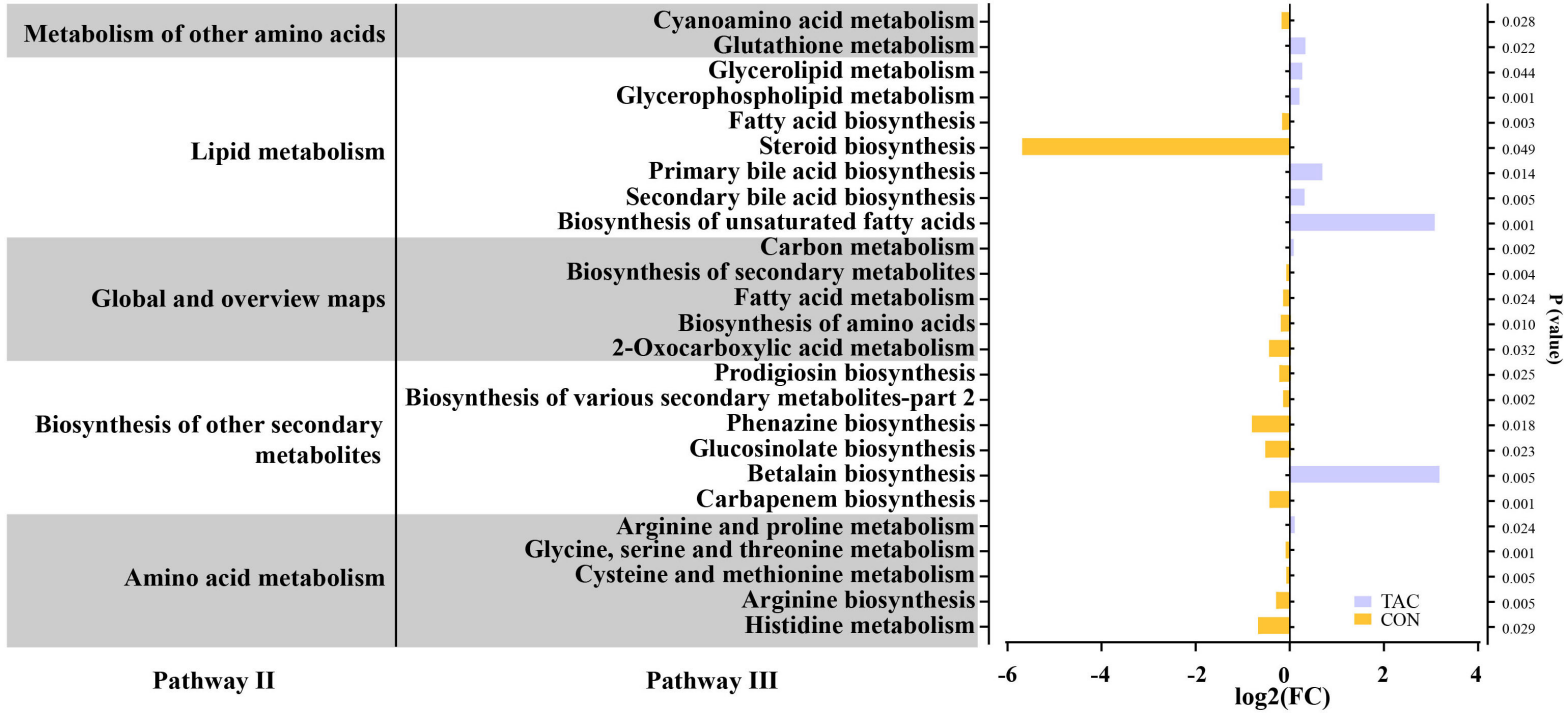
TAC-induced changes in the functional genes of the intestinal microbiota. The horizontal axis shows the relative abundance (fold changes) of differentially expressed functional genes enriched in KEGG pathways (level 3).

The functional genes in the gut microbiota were predicted with PICRUSt2. The functional genes related to metabolic pathways changed significantly in the TAC group. The TAC group had a notable decrease in the percent relative frequency of global and overview maps, amino acid metabolism, and biosynthesis of other secondary metabolites, along with a significant increase in lipid metabolism and metabolism of other AAs (**Supplementary Figure S3**). In the amino acid metabolism pathways, histidine metabolism, arginine biosynthesis, cysteine and methionine metabolism, and glycine, serine and threonine metabolism were significantly enriched in the CON group, whereas arginine and proline metabolism were significantly enriched in the TAC group. In the lipid metabolism pathways, the biosynthesis of unsaturated fatty acids, primary bile acid biosynthesis, and secondary bile acid biosynthesis were significantly enriched in the TAC group. In addition, the TAC group had significantly higher relative abundance of the biosynthesis of amino acids, fatty acid metabolism, carbon metabolism, and glutathione metabolism than the CON group (**Figure 3**).

### 3.3 Nontargeted metabolomics analysis

We further evaluated the serum metabolites in the CON and TAC groups based on nontargeted metabolomics to examine how TAC affected the glucose metabolism disorder.

**Figure 4:**
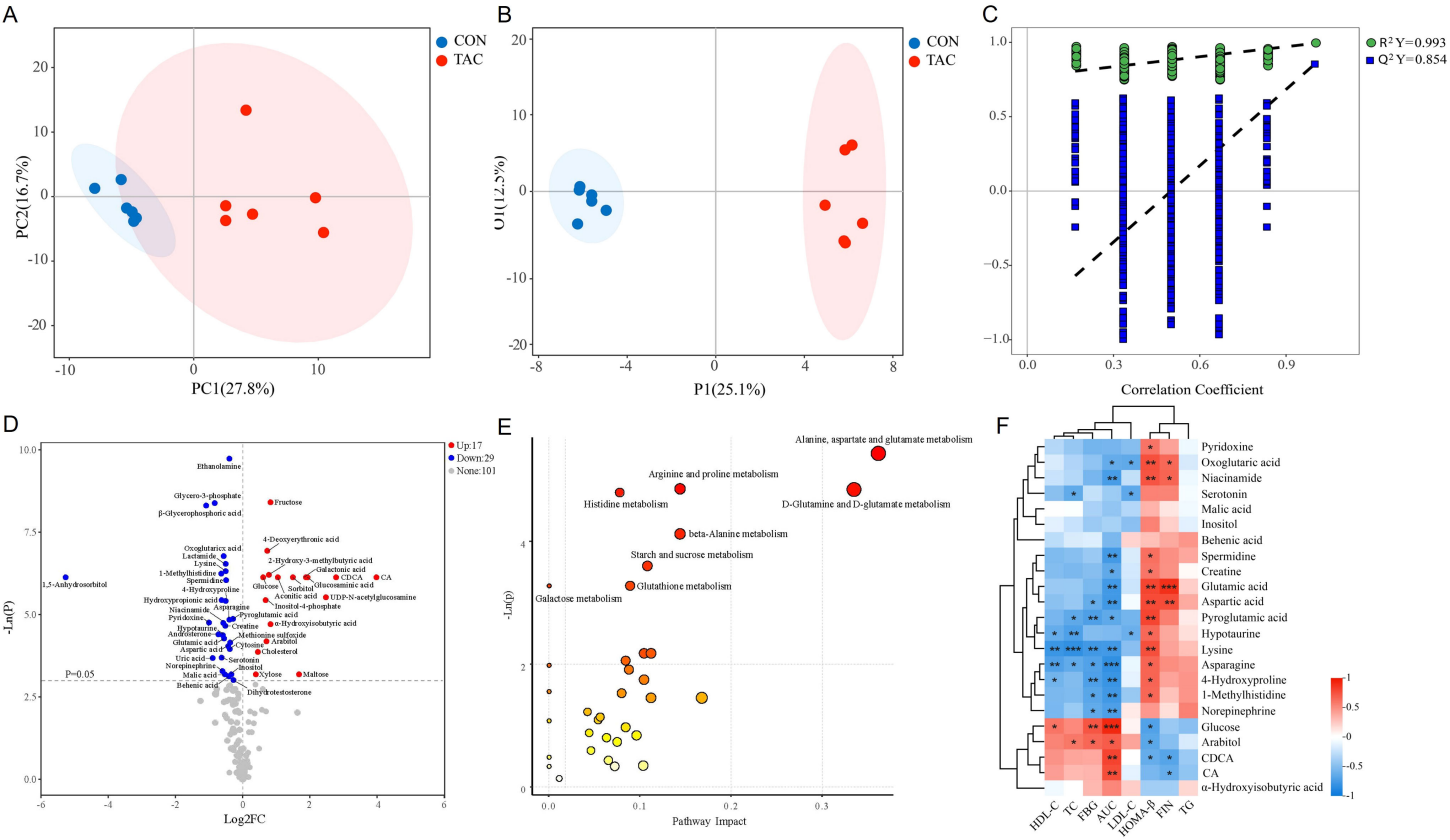
TAC induced changes in the serum metabolite profile and metabolic pathway. **(A)** PCA; **(B)** OPLS-DA scores; **(C)** 1000 random permutation test evaluation; **(D)** Volcano plot; **(E)** KEGG pathway analysis of 46 significantly different metabolites; **(F)** Heatmap of the correlation between the intestinal flora metabolites and the glycolipid metabolism indicators. *P< 0.05, **P< 0.01, ***P< 0.001.

The serum samples of the two groups were well separated in PCA and OPLS-DA (**Figures 4A, B**). The OPLS-DA permutation test (1000 times) indicated that the model exhibited good reliability and provided good prediction (*R^2^Y* = 0.993, *Q^2^Y* = 0.854) (**Figure 4C**). With the criteria VIP > 1 and *P*< 0.05, 46 common differential metabolites could be considered potential biomarkers (**Figure 4D**). Compared to the CON group, the levels of 17 metabolites, including glucosaminic acid, galactonic acid, sorbitol, CA, and CDCA, were significantly increased in the TAC group. On the other hand, the metabolites with significant decrease in the TAC group included pyroglutamic acid, asparagine, aspartic acid, 4-hydroxyproline, glutamic acid, 1-methylhistidine, and behenic acid (**Table 2**).

**Table 2:** Information on biomarkers in the serum profile.

No.	Metabolites	Database ID	RT (min)	FC	VIP	TAC/CON	Taxonomy
1	Glucosaminic acid	HMDB0341308	15.11	3.76	1.53	↑**	Carbohydrates
2	Galactonic acid	HMDB0000565	14.83	3.64	1.58	↑**	Carbohydrates
3	Maltose	HMDB0000163	20.32	3.16	1.52	↑*	Carbohydrates
4	Sorbitol	HMDB0000247	14.39	2.79	1.72	↑**	Carbohydrates
5	Fructose	HMDB0000660	13.82	1.75	1.68	↑***	Carbohydrates
6	4-Deoxyerythronic acid	HMDB0000498	8.60	1.65	1.65	↑***	Carbohydrates
7	Arabitol	HMDB0000568	12.47	1.62	1.44	↑*	Carbohydrates
8	Glucose	HMDB0000122	14.14	1.50	1.56	↑**	Carbohydrates
9	Xylose	HMDB0000098	11.92	1.29	1.31	↑*	Carbohydrates
10	1,5-Anhydrosorbitol	HMDB0002712	13.67	0.03	1.79	↓**	Carbohydrates
11	Pyroglutamic acid	HMDB0000267	10.58	0.81	1.42	↓**	Amino Acids
12	Methionine sulfoxide	HMDB0002005	13.73	0.76	1.37	↓*	Amino Acids
13	Asparagine	HMDB0000168	10.37	0.75	1.47	↓**	Amino Acids
14	Aspartic acid	HMDB0000191	10.53	0.73	1.39	↓*	Amino Acids
15	4-Hydroxyproline	HMDB0006055	10.61	0.70	1.54	↓**	Amino Acids
16	Lysine	HMDB0000182	14.31	0.70	1.56	↓**	Amino Acids
17	Creatine	HMDB0000064	10.91	0.69	1.44	↓**	Amino Acids
18	Glutamic acid	HMDB0000148	11.53	0.67	1.42	↓*	Amino Acids
19	1-Methylhistidine	HMDB0000001	13.77	0.63	1.58	↓**	Amino Acids
20	Aconitic acid	HMDB0000072	12.74	2.04	1.54	↑**	Organic Acids
21	α-Hydroxyisobutyric acid	HMDB0000729	5.25	1.80	1.35	↑**	Organic Acids
22	Malic acid	HMDB0000156	10.19	0.68	1.09	↓*	Organic Acids
23	Oxoglutaric acid	HMDB0000208	11.08	0.67	1.64	↓**	Organic Acids
24	Hypotaurine	HMDB0000965	11.37	0.66	1.32	↓*	Organic Acids
25	Hydroxypropionic acid	HMDB0000700	6.20	0.64	1.36	↓**	Organic Acids
26	Uric acid	HMDB0000289	15.68	0.53	1.31	↓*	Organic Acids
27	Cholesterol	HMDB0000067	23.19	1.35	1.29	↑*	Lipids
28	Dihydrotestosterone	HMDB0002961	19.65	0.82	1.12	↓*	Lipids
29	Androsterone	HMDB0000031	19.23	0.60	1.31	↓*	Lipids
30	Glycero-3-phosphate	HMDB0000126	12.84	0.55	1.77	↓***	Lipids
31	β-Glycerophosphoric acid	HMDB0002520	12.50	0.47	1.75	↓***	Lipids
32	Inositol-4-phosphate	HMDB0001313	17.96	1.59	1.57	↑**	Alcohols
33	Lactamide	HMDB0253942	6.19	0.69	1.55	↓**	Alcohols
34	Inositol	HMDB0000211	15.64	0.78	1.07	↓*	Alcohols
35	Ethanolamine	HMDB0000149	7.75	0.75	1.76	↓***	Amines
36	Spermidine	HMDB0001257	16.88	0.70	1.58	↓**	Amines
37	CA	HMDB0000619	24.65	15.53	1.29	↑**	Bile Acids
38	CDCA	HMDB0000518	25.26	6.75	1.44	↑**	Bile Acids
39	2-Hydroxy-3-methylbutyric acid	HMDB0000407	6.47	1.70	1.56	↑**	Fatty Acids
40	Behenic acid	HMDB0000944	19.46	0.75	1.20	↓*	Fatty Acids
41	UDP-N-acetylglucosamine	HMDB0000290	13.57	5.59	1.12	↑**	Nucleotides
42	Cytosine	HMDB0000630	10.58	0.76	1.29	↓*	Nucleotides
43	Niacinamide	HMDB0001406	10.17	0.66	1.70	↓**	Vitamins
44	Pyridoxine	HMDB0000239	14.28	0.49	1.40	↓**	Vitamins
45	Norepinephrine	HMDB0000216	16.30	0.65	1.24	↓*	Phenols
46	Serotonin	HMDB0000259	18.30	0.64	1.19	↓*	Indoles

*P< 0.05, **P< 0.01, ***P< 0.001.

The symbols ↑ and ↓ indicate an increase or decrease in the relative content of metabolites in the TAC group.

Subsequently, MetaboAnalyst was used for the pathway analysis of differential metabolites to clarify the functional changes in the serum metabolic profile in TAC-induced diabetic rats. The pathways of alanine, aspartate, and glutamate metabolism, D-glutamine and D-glutamate metabolism, arginine and proline metabolism, histidine metabolism, beta-alanine metabolism, starch and sucrose metabolism, galactose metabolism, and glutathione metabolism were significantly altered between the two groups (**Figure 4E**). Notably, arginine and proline metabolism, histidine metabolism, and glutathione metabolism also significantly differed in the functional prediction of the gut microbiota.

Metabolites associated with the microbiota, such as SCFAs, BAs, tryptophan metabolites, and phenols, have been shown to play a crucial role in the development of diabetes (Wu et al., 2023). Therefore, we screened metabolites related to the intestinal flora and analyzed their correlations with glycolipid metabolism indicators. The results indicated that BAs (CA and CDCA) and carbohydrates were positively correlated with AUC and negatively correlated with HOMA-β. Additionally, vitamins, amines, and AAs were mostly negatively correlated with FBG and AUC but positively correlated with HOMA-β (**Figure 4F**).

### 3.4 Targeted metabolomics analysis

#### 3.4.1 Serum amino acid

**Figure 5:**
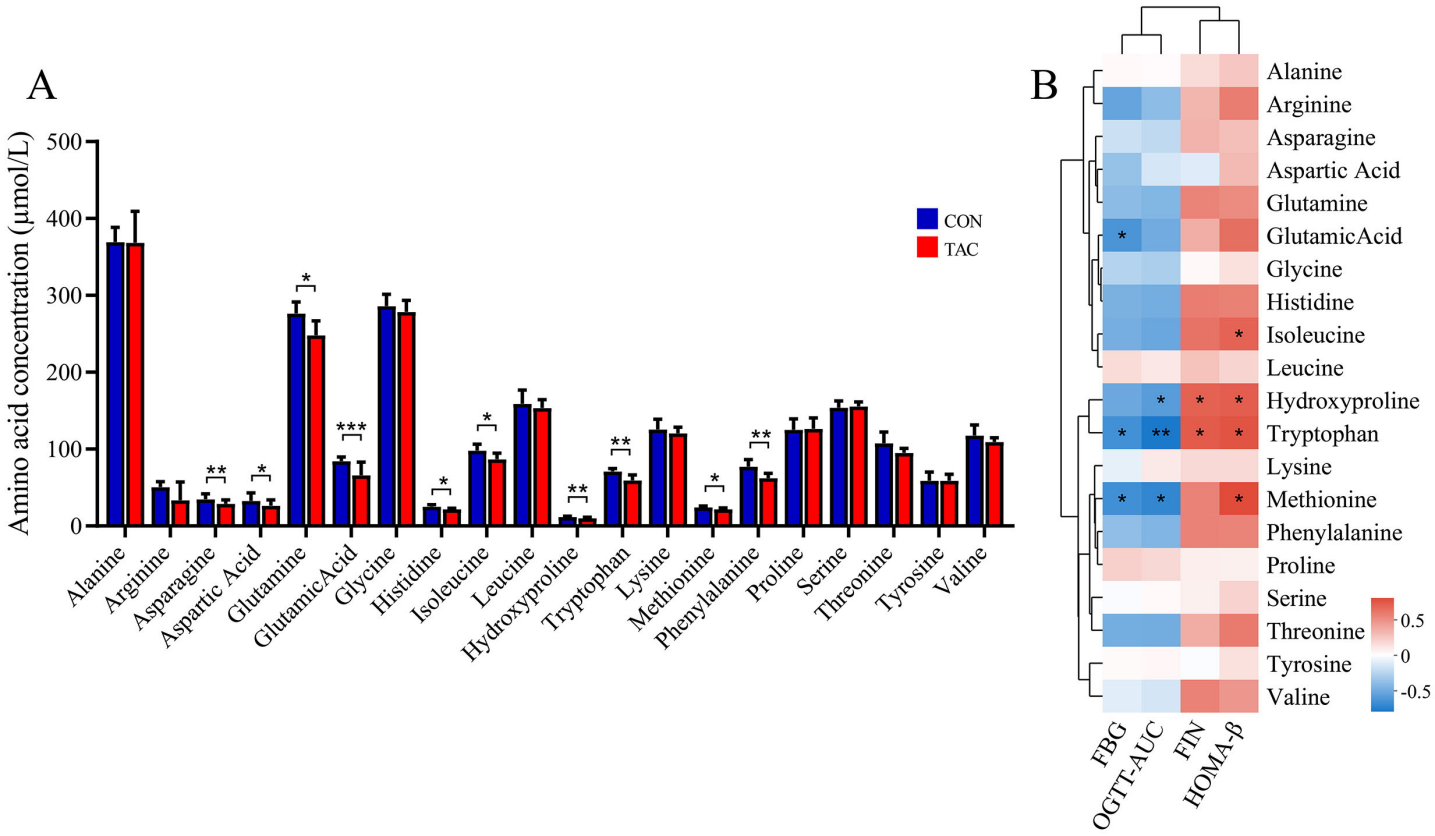
Effects of TAC on the amino acid metabolic profile. **(A)** Changes in the amino acid levels; **(B)** Correlation between amino acid and metabolic indexes. *P< 0.05, **P< 0.01, ***P< 0.001.

The TAC group showed significantly reduced serum levels of asparagine, aspartic acid, glutamine, glutamate, histidine, isoleucine, hydroxyproline, tryptophan, methionine, and phenylalanine (**Figure 5A**). The results for asparagine, aspartic acid, glutamic acid, and hydroxyproline were consistent with the nontargeted analysis, suggesting that they may play an important role in TAC-induced diabetes.

Spearman correlation was used to further determine the association between the AAs and the glucose metabolism-related indicators. The results showed that the levels of FBG and AUC of OGTT were negatively correlated with glutamic acid, hydroxyproline, tryptophan, and methionine, while FIN and HOMA-β were positively correlated with hydroxyproline, tryptophan, and tyrosine (**Figure 5B**).

#### 3.4.2 Serum and fecal bile acid

**Figure 6:**
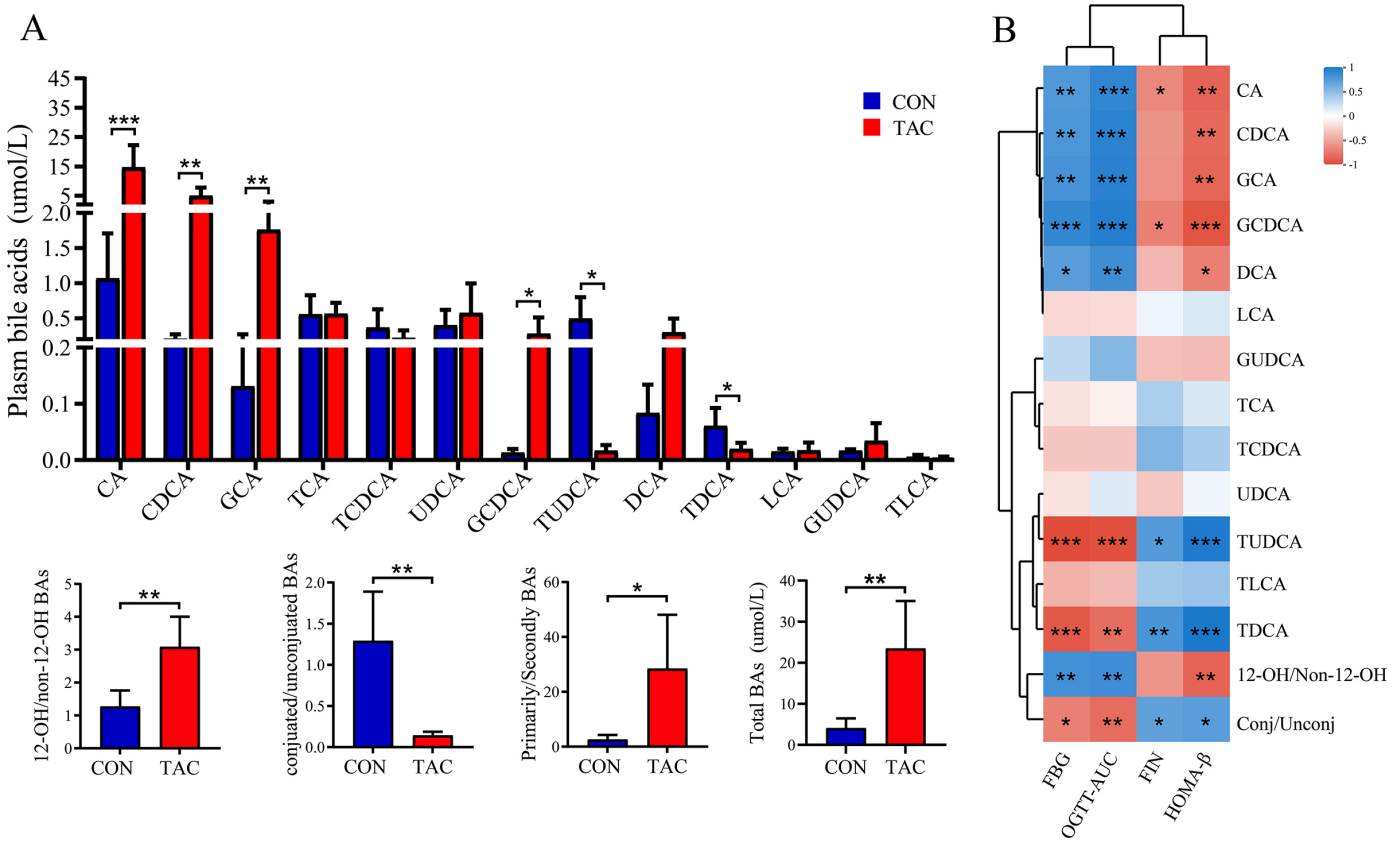
Effects of TAC on the serum bile acid metabolic profile. **(A)** Changes in bile acid levels; **(B)** Correlation between bile acids and metabolic indexes. *P< 0.05, **P< 0.01, ***P< 0.001.

The absolute quantification was carried out for 13 BAs in both serum and feces. We found that TAC significantly increased the primary/secondary BA ratio and the total BAs level, but significantly decreased the conjugated/unconjugated BAs ratio (**Figure 6A**). The TAC group had significantly higher serum levels of 12-OH/non-12-OH-BAs. Among the non-12-OH-BAs, CDCA, GCA, and GCDCA had significantly elevated levels and TUDCA and TDCA had significantly reduced levels in the TAC group. For the 12-OH-BAs, the level of CA was significantly higher in the TAC group.

The correlation between serum BAs and the index of glucose metabolism was then analyzed. Both FBG and the AUC of the OGTT were correlated significantly positively with 12-OH/non-12-OH-BAs, CA, DCA, and GCA, and significantly negatively with conjugated/unconjugated BAs, TUDCA, and TDCA. In addition, HOMA-β was negatively correlated with 12-OH/non-12-OH-BAs but positively correlated with conjugated/unconjugated BAs (**Figure 6B**).

As the gut is the site of BA metabolism, the fecal BAs were also examined. Most BAs in the feces had the same variation as in the serum. The exceptions were that the TAC group had significantly higher TCA, TCDCA, TLCA and conjugated/unconjugated BAs along with significantly lower UDCA and LCA (**Supplementary Figure S4**).

#### 3.4.3 Serum short-chain fatty acids

Short-chain fatty acids are important regulators in the glucose homeostasis (Canfora et al., 2019). There was no difference in the serum SCFAs between the two groups (**Supplementary Figure S5**).

#### 3.4.4 Correlation analysis of the gut microbiota with host metabolites

The gut microbiota carries out complex metabolic activities in the human intestine, producing numerous beneficial or harmful metabolites in the human circulatory system (Nicholson et al., 2012). Thus, Spearman’s correlation analysis was performed to identify the potential relationships between microbes (the top 50 abundant microflora at the genus level) and AA and BA-related metabolites.

**Figure 7:**
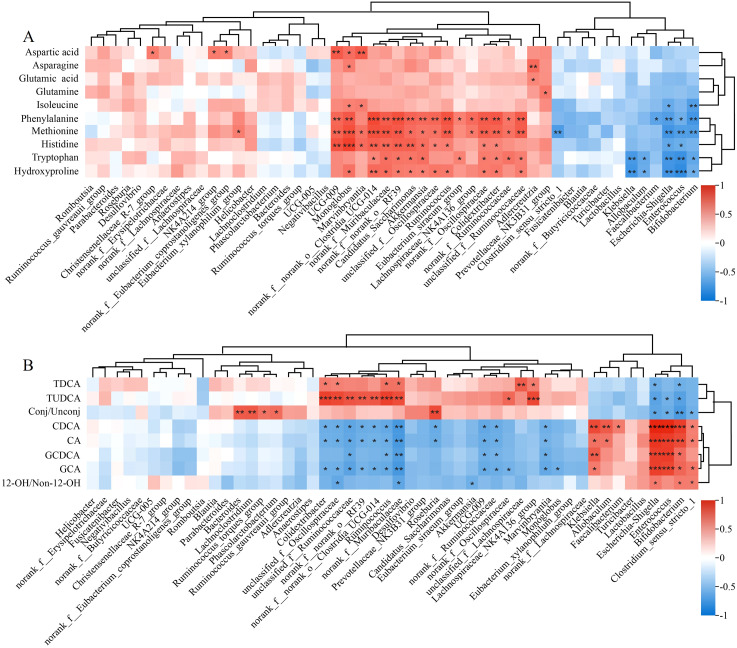
Heatmaps for the correlation between gut microbiota and metabolites related to **(A)** amino acids and **(B)** bile acids. *P< 0.05, **P< 0.01, ***P< 0.001.

A significant association existed between the changes in the gut microbiota abundance and the serum AA levels. The levels of histidine, hydroxyproline, and phenylalanine were correlated positively with the relative abundances of *Akkermansia* and *Ruminococcus* and negatively with the relative abundances of *Enterococcus* and *Bifidobacterium.* In addition, the decrease in serum glutamine was negatively correlated with *Bifidobacterium* (**Figure 7A**).

The gut microbiota was also significantly associated with serum BA levels. *Akkermansia* and *Colidextribacter* were correlated positively with CA and CDCA but negatively with TDCA and TUDCA. *Escherichia-Shigella*, *Enterococcus*, and *Bifidobacterium* were correlated positively with GCA and GCDCA. Significantly negative correlation existed between *Akkermansia* and the level of 12-OH/non-12-OH BAs (**Figure 7B**).

## 4 Discussion

In this study, we noted that the prolonged use of TAC significantly decreased the body weight of the rats, possibly due to its toxic effects (such as diabetes, hepatorenal toxicity, etc.) (Quan et al., 2020). It has been reported that TAC has a direct role in causing diabetes (Quintana-Perez et al., 2022), but the underlying mechanism has not been fully elucidated. We then used 16S rRNA sequencing and metabolomics to screen relevant biomarkers involved in TAC-induced diabetes.

The gut microbiota of TAC-induced diabetic rats had significant changes in composition at the phylum and genus levels compared to the control. After the TAC treatment, both the relative abundance of *Bacteroidetes* and the ratio of *Bacteroidetes/Firmicutes* decreased significantly. *Firmicutes* and *Bacteroidetes* are the dominant bacteria in human and animal intestine, and many studies have shown that the level of *Bacteroides* and the ratio of *Bacteroides/Firmicutes* are negatively correlated with blood glucose levels (Tilg and Moschen, 2014; Gurung et al., 2020). The negative correlation between *Bacteroides/Firmicutes* and FBG was also observed in our TAC-treated rats (r = −0.763, *P* = 0.004). At the genus level, the TAC-induced diabetic rats had higher abundances of *Escherichia-Shigella* and *Allobaculum* and lower abundances of *Ruminococcus*, *Akkermansia*, and *Roseburia*. Zhang et al. also reported previously that for the mice after 14 days of TAC administration, the relative abundances increased significantly for *Allobaculum* and decreased significantly for *Clostridium*, *Ruminococcus*, and *Oscillospira* in the gut microbiota (Zhang et al., 2018). Meanwhile, we found that the changes in *Ruminococcus*, *Roseburia*, and *Akkermansia* were closely related to FBG and FIN levels according to the correlation analysis. It has been reported that *Roseburia* and *Akkermansia* help to restore insulin sensitivity and improve glucose metabolism, and a decrease in *Ruminococcus* abundance may be related to changes in the blood glucose of the host (Huda et al., 2021). Another study suggested that the decreased abundance of *Roseburia* may be directly related to the development of T2DM (Karlsson et al., 2013). These results suggested that changes in the structure of the gut microbiota may be related to diabetes induced by TAC.

With the development of metagenomics and metabolomics, it has been found that small molecular compounds metabolized by the gut microbiota can act as a “bridge” between the gut microbiota and disease (Koh and Backhed, 2020). Therefore, we identified the differences in a variety of metabolites, including AAs, lipids, amines, BAs, and fatty acids, between the CON and TAC groups through non-targeted metabolomics. The interactions between these metabolites and the cell receptors may impact the health of the host (Massey and Brown, 2021).

Amino acids play an important role in the development of T2DM and IR (Ding et al., 2023). After TAC exposure, the pathways related to AA metabolism (alanine, aspartic acid, and glutamic acid metabolism, D-glutamine and D-glutamic acid metabolism, arginine and proline metabolism, histidine metabolism) in the serum of SD rats were interfered. This was further confirmed by the decreasing serum levels of aspartic acid, glutamic acid, glutamine, histidine, and hydroxyproline in targeted metabolomics analysis. Low levels of asparagine and aspartic acid are associated with an increased risk of T2DM (Ottosson et al., 2018; Chen et al., 2021). A meta-analysis of metabolomics studies revealed that higher levels of glutamine and glutamate in the blood are associated with a lower risk of diabetes (Guasch-Ferre et al., 2016). Our results revealed that glutamate levels were negatively correlated with FBG, and the TAC group had lower glutamate levels. Glutamate can be converted to glutathione, and oxidative stress caused by glutathione depletion plays an important role in the development of T2DM (Lv et al., 2023). The reduction of insulin resistance is also intimately associated with histidine and its metabolites (Holecek, 2020). Because insulin resistance (including the inhibition of proteolysis) can increase the release of BCAAs in muscle and increase their circulating levels (Mccormack et al., 2013), high levels of plasma BCAAs (leucine, isoleucine, and valine) may be associated with a high risk of PTDM in renal transplant recipients (Oste et al., 2020). However, animal studies have found that supplementation of BCAAs in mice receiving a high fat diet can promote insulin action and signal transduction (Macotela et al., 2011). These conflicting results suggest that the relationship between BCAAs and the occurrence and development of T2DM is complex. Our targeted metabolomics analysis showed that the serum isoleucine levels were significantly reduced by the TAC treatment. Clinical studies have also found that serum leucine levels were significantly reduced in renal transplant patients after TAC treatment (Kim et al., 2010), which may be related to the increase of BCAAs aminotransferase activity in the renal parenchyma and the depletion of BCAAs caused by the decrease of branched-chain α-ketoacid dehydrogenase activity (Adeva et al., 2012; Saleem et al., 2019). The above results suggested that AAs may be a potential marker for TAC-induced diabetes. Previous research has also shown that supplementing mice with a BCAA mixture can increase the abundances of *Akkermansia* and *Bifidobacterium* and decrease the abundance of *Enterobacteriaceae* in the gut microbiota (Yang et al., 2016). In addition, studies have indicated that for mice having a high-fat diet, when the diet was supplemented with resistant dextrin, the levels of bacteria such as *Paraclactis*, *Brautella*, and *Dubosiella* in the gut microbiota were also changed, which consequently affected the levels of their metabolites, including BAs (enterohepatic circulation), indole derivatives (tryptophan metabolism), and lipoic acid (lipid metabolism) (Zhang et al., 2021). Tryptophan is an essential amino acid that is produced through protein fermentation in food. It is converted into indole and its derivatives by bacteria in the large intestine and then utilized by the body. Indole derivatives are microbial tryptophan catalysts beneficial to human health. A higher circulating indole propionate is associated with a lower risk of T2DM (Tuomainen et al., 2018). Tryptophanase, which can decompose tryptophan to produce indole, exists in the intestinal bacteria such as *Bacteroides polymorphus*, *Clostridium ovalis*, *Clostridium citricum*, *Enterococcus faecalis*, and *Escherichia coli*. The protective effect and mechanism of these metabolites on diabetes require further exploration and verification (Lee and Lee, 2010; Gao et al., 2023).

Significant changes in specific BAs species were found in this study. In the TAC rats, the serum levels of CA, CDCA, GCA, TCA, GCDCA, and DCA increased significantly, whereas the levels of TDCA and TUDCA decreased significantly. As the main component of bile, primary BAs are synthesized in the liver based on cholesterol by a series of enzymatic reactions and are excreted into the intestine through the gallbladder. They are also bio-transformed in the colon to secondary BAs by the gut microbiota, and both primary and secondary BAs can be returned to the liver upon reabsorption in the ileum and colon (Chiang, 2013; Di Ciaula et al., 2017). Over the last few decades, BAs have attracted considerable attention in the integrated regulation of lipid, glucose, energy metabolism, etc. However, current clinical studies have shown conflicting results. For instance, Sonne et al. found that T2DM patients had higher postprandial total BAs, CA, CDCA, DCA, and UDCA levels than healthy patients (Sonne et al., 2014). In contrast, a Chinese cohort study showed that the T2DM risk was associated negatively with the uncombined BAs (CA, CDCA, and DCA), although it was associated positively with the combined primary BAs (GCA, TCA, GCDCA, and TCDCA) and with secondary BAs (TUDCA) (Lu et al., 2021). In addition, in our study, the administration of TAC significantly increased the serum and fecal 12-OH/non-12OH BAs. Studies have suggested 12-OH/non-12-OH BAs ratio as a potential biomarker for hyperglycemia and insulin resistance, since a higher ratio is associated with islet signaling, higher blood glucose, and lower insulin sensitivity (Haeusler et al., 2012; Haeusler et al., 2013; Qi et al., 2015; Zhong et al., 2022). We also found that the serum BA levels were closely related to FBG, AUC of OGTT, and FIN. It has been discovered that intestinal bacteria, such as *Bacteroides*, *Bifidobacterium*, *Clostridium*, *Lactobacillus*, and *Ruminococcus*, play a crucial role in the metabolism of BAs (Okekunle et al., 2019). A key step in BA biotransformation is the microbial debinding by bile acid hydrolase (BSH). Intestinal bacteria including *Lactobacillus*, *Bifidobacterium*, *Enterococcus*, and *Bacteroides* all exhibit BSH activity (Wahlstrom et al., 2016). Previous studies have demonstrated that antioxidants can affect BSH activity and lead to changes in BA composition to ultimately ameliorate obesity (Huang et al., 2019). Primary BAs that are not reabsorbed enter the colon and are metabolized to secondary BAs by 7-dehydroxylation. For example, CA and CDCA are metabolized to DCA and LCA respectively. The 7-dehydroxylation process involves multiple reactions between *Firmicutes* (mainly *Clostridium* and *Eubacterium genera*) and genes induced by BAs (Collins et al., 2023). Furthermore, recent studies have indicated that changes in BA levels may be associated with bacteria rich in β-glucuronidase (GUS) (Li et al., 2024). A significant increase in the intestinal GUS activity was found in TAC-induced diabetic mice. On the contrary, *Bacteroides* play a pivotal role in the synthesis of GUS in intestinal microbes and the impact of GUS on drug glucuronidation (Elmassry et al., 2021). Therefore, further research is necessary to validate the significance of gut microbiota and BAs in TAC-induced diabetes.

SCFAs are mainly produced by the fermentation of dietary fiber in food by probiotics (Louis et al., 2014). The regulation of blood glucose by SCFAs has received extensive attention. In T2DM patients, reduced SCFA levels caused by a decreased abundance of SCFA-producing bacteria are associated with insulin resistance and the development of T2DM (Zhao et al., 2018). In mice with diabetes, butyrate supplementation could promote glycogen storage in hepatocytes and improve blood glucose and hepatic glycogen metabolic homeostasis (Zhang et al., 2019). Jiao et al. noted the decrease of SCFAs, especially butyrate, in TAC-induced diabetic mice, and found that butyrate supplementations in the diet significantly improved the glucose tolerance of TAC-induced diabetic mice and increased the expression of GLP-1 in colon and GPR43 in the intestinal crypt (Jiao et al., 2020). However, in our experiments, we observed no difference in SCFAs between the CON group and the TAC group. The observed lack of difference may be attributed to the fact that TAC primarily exerts its effects on the gut microbiota (Wang et al., 2023).

This study only has omics data and approximate correlation analysis. Additional research is needed to verify if the identified metabolites and bacteria have the same trends in clinical findings. Another limitation of this work is that it does not experimentally confirm any causal relationship between intestinal microbiota and host diseases, and relevant experimental evidence is needed in the future.

## 5 Conclusions

We for the first time evaluated the changes of the gut microbiota and the host metabolism in TAC-induced diabetic rats. The analyses showed that the alterations in the gut microbiota and metabolites, particularly *Ackermannia*, *Ruminococcus*, *Roseburia*, BAs, and AAs, may participate in the progression of TAC-induced diabetes. However, no conclusive evidence could be recognized to suggest that TAC leads to diabetes via the changes in microbial metabolites. The role of microbial metabolites in the development of TAC-induced diabetes remains to be further studied.

## Data Availability

The raw sequencing data in our study have been deposited in the BioProject, accession number: PRJNA1157748.
