# Supplementary material for: Gut microbiota and metabolomic profile changes play critical roles in tacrolimus-induced diabetes in rats
Source: Front Cell Infect Microbiol. 2024 Sep 17;14:1436477. doi: 10.3389/fcimb.2024.1436477 (PMC11442430; doi:10.3389/fcimb.2024.1436477)
Supplement: Supplementary file 1 [file Table1.docx]

Supplementary Material

# BAs, AAs and SCFAs chromatographic and mass spectrometry conditions

## BAs LC-MS/MS analysis

The AB SCIEX Triple Quad 4500MD mass spectrometer connected to a Jasper HPLC system (Applied Biosystem Sciex, Toronto, ON, Canada) was used in this method. The analytes were separated using a Kinetex EVO C18 column (50×2.1 mm, 2.6 μm, Phenomenex, United States) with a two-solvent gradient system at a column temperature of 40 ℃. The mobile phase consists of 0.1% formic acid and 0.5% ammonia (A) -acetonitrile (B). The injection volume was 10 μL. The gradient elution procedure was eluted at a flow rate of 0.4 mL/min with the following gradient program in Table S1:

**Table S1** Gradient elution procedure of BAs

| **Time (min)** | **Flow (mL/min)** | **A%** | **B%** |
| --- | --- | --- | --- |
| 0.0 | 0.4 | 75 | 25 |
| 4.5 | 0.4 | 65 | 35 |
| 6.0 | 0.4 | 50 | 50 |
| 6.1 | 0.4 | 5 | 95 |
| 7.4 | 0.4 | 5 | 95 |
| 7.5 | 0.4 | 75 | 25 |
| 9.0 | 0.4 | 75 | 25 |

The MS/MS detection was accomplished using multiple reaction monitoring (MRM) in electrospray negative ionization mode. The optimized MRM conditions for all analytes are presented in Table S2. Other parameters were set as follows: capillary voltage, 5.5 kV; source temperature, 500 C; curtain gas pressure, 30 psi; ion spray voltage, 5000 V; Ion source gas 1/2, 55 psi; and collision gas pressure, 8 psi. High-purity nitrogen served as both the nebulizing and drying gas.

**Table S2** Mass spectrum conditions of BAs

| **Analytes** | **Precursor ion (Da)** | **Product ion (Da)** | **Declustering potential (V)** | **Collision energy (V)** | **Retention time (min)** |
| --- | --- | --- | --- | --- | --- |
| CA | 407.1 | 407.1 | -20 | -165 | 1.25 |
| GCA | 464.3 | 74.1 | -78 | -150 | 1.51 |
| GUDCA | 448.3 | 74.0 | -70 | -150 | 1.30 |
| TCA | 514.2 | 80.0 | -115 | -210 | 1.76 |
| TUDCA | 498.3 | 498.3 | -35 | -210 | 1.60 |
| UDCA | 391.3 | 391.3 | -25 | -165 | 1.10 |
| CDCA | 391.3 | 391.3 | -24 | -160 | 2.30 |
| GCDCA | 448.3 | 74.0 | -72 | -150 | 2.75 |
| TCDCA | 498.3 | 498.3 | -35 | -200 | 3.10 |
| TDCA | 498.3 | 79.8 | -115 | -210 | 3.42 |
| DCA | 391.3 | 391.3 | -25 | -165 | 2.68 |
| LCA | 375.3 | 375.3 | -35 | -190 | 4.32 |
| TLCA | 482.2 | 79.9 | -115 | -210 | 5.24 |
| GCA-d5 | 469.3 | 469.3 | -15 | -150 | 1.51 |
| CDCA-d4 | 395.3 | 395.3 | -24 | -180 | 2.30 |
| GCDCA-d7 | 455.3 | 75.0 | -70 | -150 | 2.75 |
| DCA-d5 | 396.3 | 396.3 | -25 | -165 | 2.68 |
| LCA-d4 | 379.3 | 379.3 | -30 | -180 | 4.32 |

## AAs LC-MS/MS analysis

The ExionLC AD UPLC system was used in this method. The analytes were separated using a Waters BEH Amide column (100×2.1 mm, 1.7 μm) with a two-solvent gradient system. Mobile phase A (0.4% formic acid 20mM ammonium formate-95% acetonitrile solution), mobile phase B (0.4% formic acid 20mM ammonium formate-5% acetonitrile solution). The injection volume was 2 μL. The gradient elution procedure was shown in Table S3:

**Table S3** Gradient elution procedure of AAs

| **Time (min)** | **Flow (mL/min)** | **A%** | **B%** |
| --- | --- | --- | --- |
| 0.0 | 1 | 100 | 0 |
| 1.0 | 1 | 90 | 10 |
| 2.6 | 1 | 85 | 15 |
| 3.5 | 1 | 70 | 30 |
| 4.0 | 1 | 70 | 30 |
| 4.1 | 1 | 100 | 0 |
| 6.0 | 1 | 100 | 0 |

The MS/MS detection was accomplished using AB SCIEX QTRAP 6500+ mass spectrometer combined with a multiple reaction monitoring (MRM) in electrospray positive ionization mode. The parameters were set as follows: curtain gas pressure, 35 psi; collision gas, medium; ion spray voltage, 5500 V; source temperature, 350 C; Ion source gas 1/2, 70 psi. The standard ion pair parameter informations are presented in Table S4.

**Table S4** Mass spectrum conditions of AAs

| **Analytes** | **Precursor ion (Da)** | **Product ion (Da)** | **Declustering potential (V)** | **Collision energy (V)** | **Retention time (min)** |
| --- | --- | --- | --- | --- | --- |
| Alanine | 90.0 | 44.1 | 10 | 16 | 3.08 |
| Arginine | 175.1 | 70.1 | 50 | 32 | 4.06 |
| Asparagine | 133.1 | 74.1 | 25 | 23 | 3.64 |
| Aspartic Acid | 134.0 | 88.2 | 10 | 14 | 3.81 |
| Cystine | 241.1 | 73.9 | 50 | 24 | 3.32 |
| Glutamine | 147.1 | 84.0 | 20 | 24 | 3.58 |
| Glutamic Acid | 148.1 | 84.0 | 30 | 24 | 3.55 |
| Glycine | 76.1 | 30.0 | 10 | 18 | 3.37 |
| Histidine | 156.1 | 110.0 | 50 | 20 | 4.07 |
| Isoleucine | 132.0 | 86.1 | 10 | 14 | 2.21 |
| Cysteine | 122.1 | 59.1 | 5 | 35 | 2.74 |
| Leucine | 132.1 | 86.1 | 10 | 15 | 2.09 |
| Hydroxyproline | 132.1 | 86.1 | 60 | 25 | 3.14 |
| Tryptophan | 205.1 | 146.1 | 80 | 26 | 2.03 |
| Lysine | 147.1 | 84.0 | 30 | 25 | 4.14 |
| Methionine | 150.2 | 104.0 | 10 | 15 | 2.39 |
| Phenylalanine | 166.1 | 120.1 | 60 | 21 | 2.04 |
| Proline | 116.1 | 70.2 | 30 | 20 | 2.51 |
| Serine | 106.0 | 60.1 | 20 | 16 | 3.59 |
| Threonine | 120.1 | 74.2 | 15 | 15 | 3.31 |
| Tyrosine | 182.2 | 136.0 | 30 | 20 | 2.54 |
| valine | 118.1 | 55.1 | 50 | 25 | 2.49 |

## SCFAs LC-MS/MS analysis

LC-ESI-MS/MS (UHPLC-Qtrap) was used to conduct qualitative and quantitative detection of the target in the sample. The specific parameters are as follows:

The AB SCIEX QTRAP 6500+ mass spectrometer connected to a ExionLC AD UPLC system was used in this method. The analytes were separated using a Waters BEH C18 column (150×2.1 mm,1.7 μm) at a column temperature of 40 ℃. The mobile phase consists of 0.1% formic acid-aqueous (A) -0.1% formic acid-acetonitrile (B). The injection volume was 2 μL. The gradient elution procedure were eluted at a flow rate of 0.35 mL/min with the following program (Table S5):

**Table S5** Gradient elution procedure of SCFAs

| **Time (min)** | **Flow (mL/min)** | **A%** | **B%** |
| --- | --- | --- | --- |
| 0.0 | 0.35 | 90 | 10 |
| 2.0 | 0.35 | 90 | 10 |
| 11.0 | 0.35 | 45 | 55 |
| 12.0 | 0.35 | 5 | 95 |
| 13.0 | 0.35 | 5 | 95 |
| 13.1 | 0.35 | 90 | 10 |
| 16.0 | 0.35 | 90 | 10 |

The MS/MS detection was accomplished using multiple reaction monitoring (MRM) in electrospray negative ionization mode. The optimized MRM conditions for all analytes are presented in Table S6. Other parameters were set as follows: curtain gas pressure, 35 psi; collision gas, medium; ion spray voltage, -4500 V; source temperature, 450 C; Ion source gas 1/2, 40 psi.

**Table S6** Mass spectrum conditions of SCFAs

| **Analytes** | **Precursor ion (Da)** | **Product ion (Da)** | **Declustering potential (V)** | **Collision energy (V)** | **Retention time (min)** |
| --- | --- | --- | --- | --- | --- |
| Acetic acid | 194 | 137 | -20 | -22 | 6.2 |
| Propanoic acid | 208 | 137 | -60 | -24 | 7.49 |
| Isobutyric acid | 222 | 137 | -80 | -24 | 8.64 |
| Butanoic acid | 222 | 137 | -80 | -24 | 8.8 |
| Isovaleric acid | 236 | 137 | -90 | -27 | 9.92 |
| Valeric acid | 236 | 137 | -90 | -27 | 10.16 |
| Isohexanoic acid | 250 | 137 | -100 | -30 | 11.26 |
| Hexanoic acid | 250 | 137 | -100 | -30 | 11.4 |

**Table S7** Comparison of α diversity index between groups.

|  | **ACE index** | **Chao 1 index** | **Shannon index** | **Simpson index** |
| --- | --- | --- | --- | --- |
| CON | 600.59 ± 82.38 | 607.38 ± 84.28 | 3.88 ± 0.39 | 0.06 ± 0.02 |
| TAC | 424.37 ± 187.42 | 425.99 ± 192.61 | 2.98 ± 0.85 | 0.12 ± 0.07 |
| *P*-Value | ns | ns | ns | ns |

ns：CON vs. TAC; There were no statistical difference.

# Supplementary Figures

**
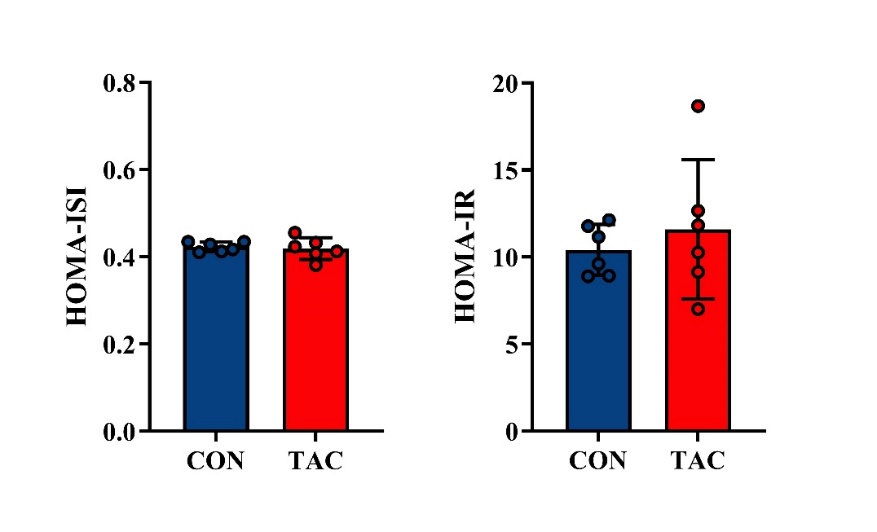
**

**Supplementary Figure 1.** **Effect of TAC on SD rats (A) HOMA-ISI and (B) HOMA-IR.**

**
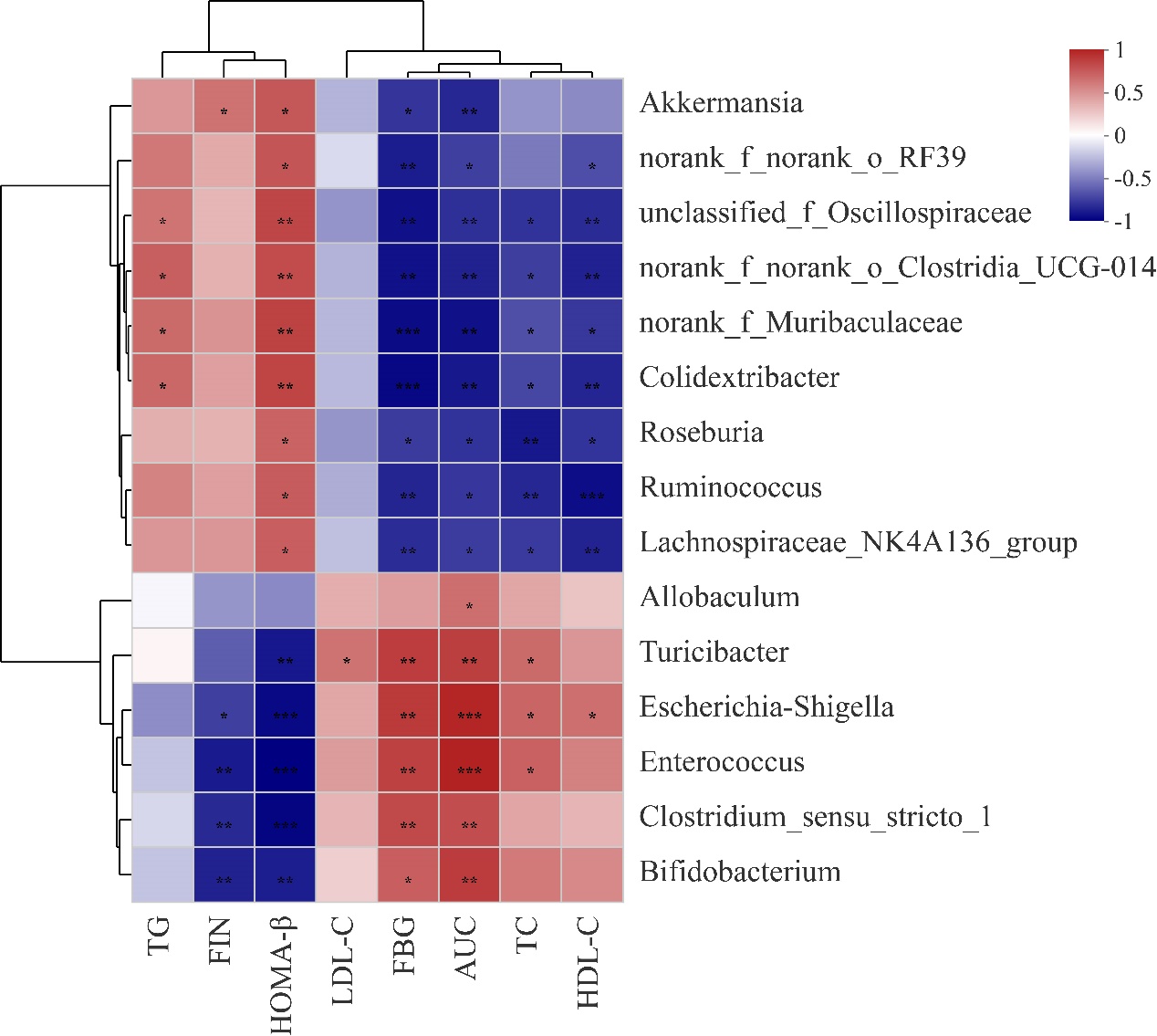
**

**Supplementary Figure 2. Correlation analysis of different gut microbiota and physiological indexes. *p<0.05, **p<0.01, ***p<0.001.**

**
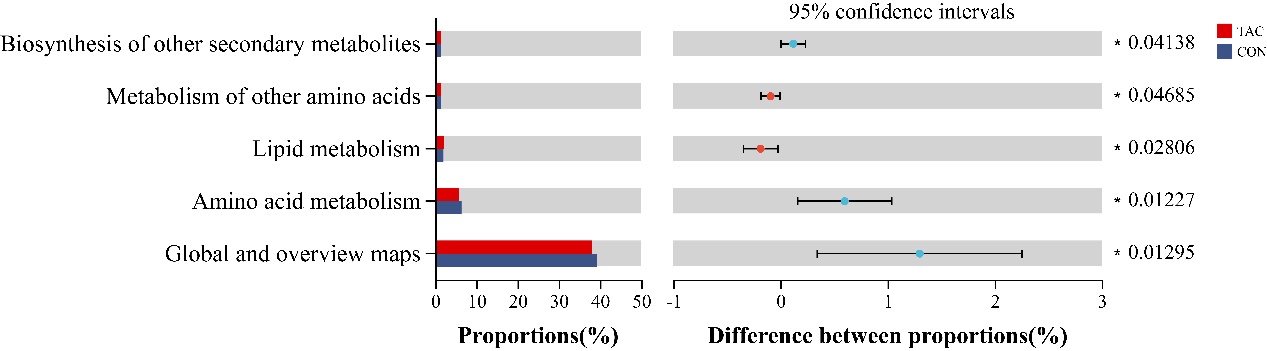
**

**Supplementary Figure 3. The relatively abundant pathways of microbial genes between the two groups at the pathway II level. *p<0.05.**

**
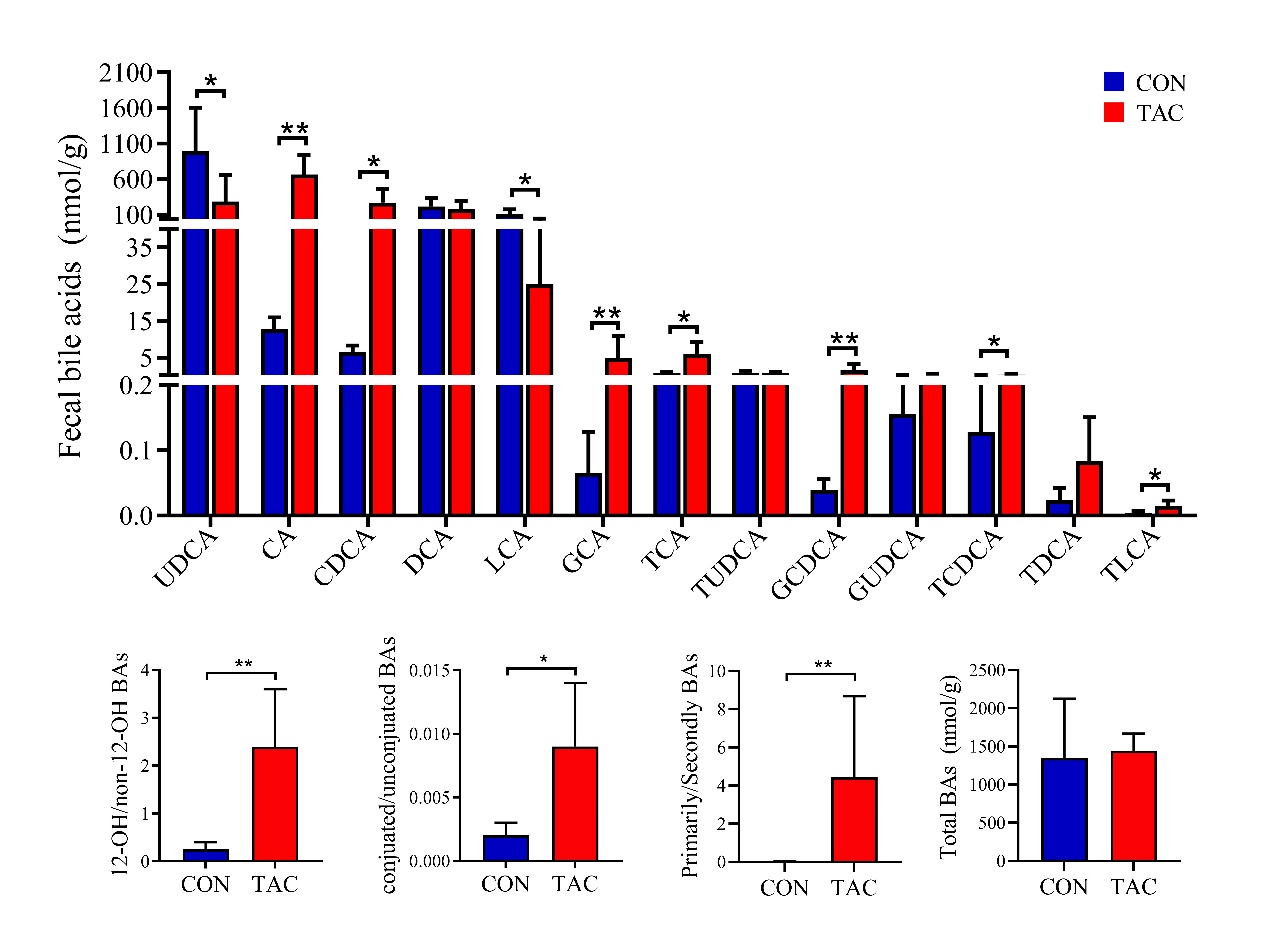
**

**Supplementary Figure 4. Fecal bile acid concentration in the TAC and CON groups. *p<0.05, **p<0.01.**

**
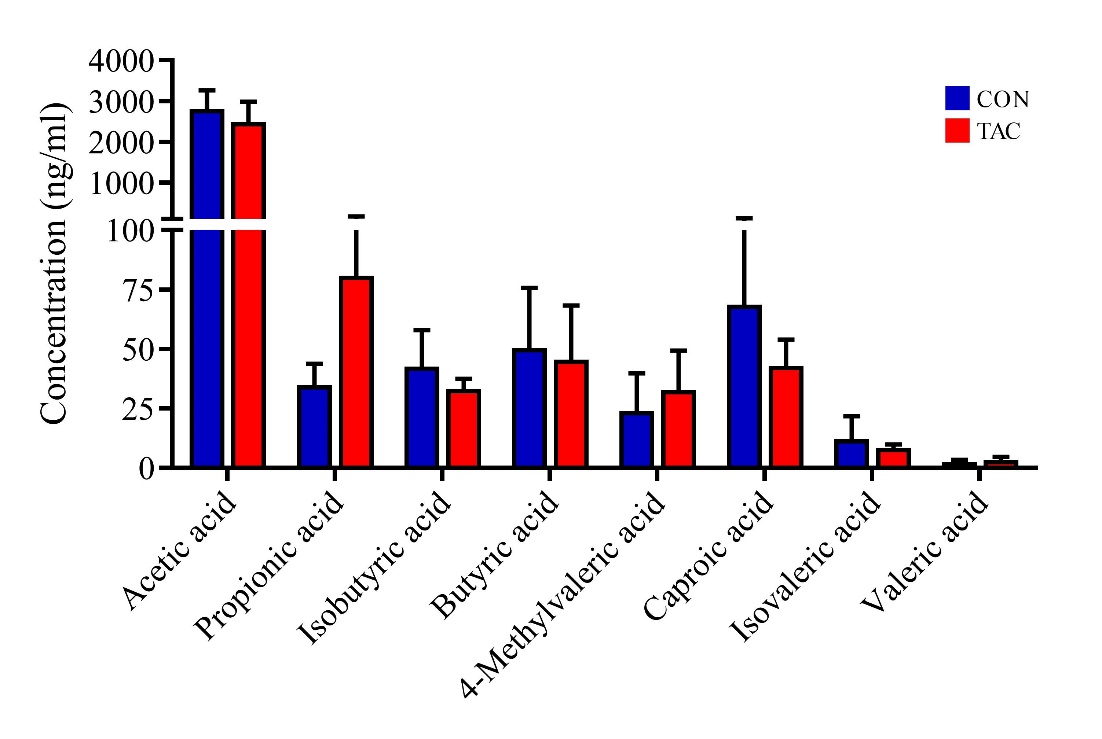
**

**Supplementary Figure 5. Serum SCFAs concentration in the TAC and CON groups. There was no significant difference between the two groups.**
